# RIPK2: New Elements in Modulating Inflammatory Breast Cancer Pathogenesis

**DOI:** 10.3390/cancers10060184

**Published:** 2018-06-05

**Authors:** Alaa Zare, Alexandra Petrova, Mehdi Agoumi, Heather Amstrong, Gilbert Bigras, Katia Tonkin, Eytan Wine, Shairaz Baksh

**Affiliations:** 1 Department of Pediatrics, Faculty of Medicine and Dentistry, University of Alberta, 113 Street 87 Avenue, Edmonton, AB T6G 2E1, Canada; zare.alaa@gmail.com (A.Z.); harmstro@ualberta.ca (H.A.); wine@ualberta.ca (E.W.); 2 Department of Biochemistry, Faculty of Medicine and Dentistry, University of Alberta, 113 Street 87 Avenue, Edmonton, AB T6G 2E1, Canada; apetrova@ualberta.ca; 3 Anatomic Pathologist at DynalifeDx, Diagnostic Laboratory Services; Department of Laboratory Medicine and Pathology, University of Alberta, 113 Street 87 Avenue, Edmonton, AB T6G 2R3, Canada; mehdi.agoumi@dynalifedx.com; 4 Cross Cancer Institute Department of Laboratory Medicine and Pathology, University of Alberta, 11560 University Ave, Edmonton, AB T6G 1Z2, Canada; gilbert.bigras@albertahealthservices.ca; 5 Division of Medical Oncology, Department of Oncology, Faculty of Medicine and Dentistry, University of Alberta, Edmonton, AB T6G 2R7, Canada; katiaton@yahoo.com; 6 Division of Experimental Oncology, Department of Oncology, Faculty of Medicine and Dentistry, University of Alberta, 113 Street 87 Avenue, Edmonton, AB T6G 2E1, Canada; 7 Cancer Research Institute of Northern Alberta, University of Alberta, Edmonton, AB T6G 2R7, Canada; 8 Women and Children’s Health Research Institute, Edmonton Clinic Health Academy (ECHA), University of Alberta, 4-081 11405 87 Avenue NW Edmonton, AB T6G 1C9, Canada

**Keywords:** IBC, inflammation, NF-κB, RIPK2, NOD2, RASSF1A, metastasis

## Abstract

Inflammatory breast cancer (IBC) is a rare and aggressive form of breast cancer that is associated with significantly high mortality. In spite of advances in IBC diagnoses, the prognosis is still poor compared to non-IBC. Due to the aggressive nature of the disease, we hypothesize that elevated levels of inflammatory mediators may drive tumorigenesis and metastasis in IBC patients. Utilizing IBC cell models and patient tumor samples, we can detect elevated NF-κB activity and hyperactivation of non-canonical drivers of NF-κB (nuclear factor kappaB)-directed inflammation such as tyrosine phosphorylated receptor-interacting protein kinase 2 (pY RIPK2), when compared to non-IBC cells or patients. Interestingly, elevated RIPK2 activity levels were present in a majority of pre-chemotherapy samples from IBC patients at the time of diagnosis to suggest that patients at diagnosis had molecular activation of NF-κB via RIPK2, a phenomenon we define as “molecular inflammation”. Surprisingly, chemotherapy did cause a significant increase in RIPK2 activity and thus molecular inflammation suggesting that chemotherapy does not resolve the molecular activation of NF-κB via RIPK2. This would impact on the metastatic potential of IBC cells. Indeed, we can demonstrate that RIPK2 activity correlated with advanced tumor, metastasis, and group stage as well as body mass index (BMI) to indicate that RIPK2 might be a useful prognostic marker for IBC and advanced stage breast cancer.

## 1. Introduction

Inflammatory breast cancer (IBC) is a rare and aggressive form of breast cancer that is associated with high mortality. It is associated with a pT4d pathological stage due to the presence of clinical skin changes with or without histological demonstration of lymphatic involvement and corresponds to a clinical stage IIIB or worse [1]. IBC accounts for approximately 5% of all breast cancers [2,3,4] but 10% of all breast cancer deaths [5] with an approximately 35–40% five-year survival rate [6]. The term “inflammatory” refers to the clinical skin manifestations of the disease, which includes breast edema, erythema, and peau d’orange involving at least 1/3 of the skin surface of the breast caused by dermal lymphatics emboli [7,8]. Approximately 50% of IBC patients lack a tumor mass that can be felt during physical examination or that can be seen on mammogram, making IBC hard to diagnose using standard radiological investigations [9,10]. Therefore, the National Cancer Institute has set minimum criteria for the diagnosis of IBC, which includes breast inflammation symptoms (aforementioned) lasting for less than six months, erythema covering at least a third of the breast, and breast biopsy showing invasive carcinoma. Despite improvements in diagnostic imaging using ultrasound, computed tomography, or positron emission tomography modalities, IBC is still largely diagnosed based on clinical criteria with no specific validated molecular criteria. In addition, little is known about the cellular mechanisms underlying the aggressiveness of this disease [11,12] and what drives metastasis in one out of three IBC patients [13,14]. Thus, understanding the biologic and molecular characteristics of IBC should aid in earlier diagnosis and improved treatment of the disease.

IBC is an aggressive subtype of breast cancer whereby the associated “molecular inflammation” is a main player in driving both tumorigenesis and metastasis. We define “molecular inflammation” as activation of inflammatory mediators but with no overt pathological inflammation as observed in inflammatory bowel disease or arthritis. It has been established over the past decade that inflammation is a major driver of neoplastic transformation in many cancers through modulating transcription factor activity, cytokine and chemokine production, cell proliferation, apoptosis inhibition, angiogenesis, and metastases promotion [15,16]. NF-κB is a key transcription factor important in inflammation [16] and malignant transformation [17]. Upregulation of NF-κB promotes proliferation, invasiveness, metastasis and anti-apoptosis of cancer cells [18,19,20]. cDNA microarray results and gene expression profiling of IBC patient samples indicate up-regulation of NF-κB related cytokines and overexpression of NF-κB target genes such as IL-8 and VEGF (vascular endothelial growth factor), signifying that NF-κB is constitutively active in IBC patients [19,20,21,22,23].

NF-κB can be activated by cytosolic microbial receptors such as NLRs (nucleotide binding and oligomerization domain (NOD)-like receptors), which include NOD2 (nucleotide-binding oligomerization domain) [24]. NOD2 is activated by MDP (muramyl dipeptide), a natural component of gram positive and gram negative bacteria [25]. Upon NOD2 activation, RIPK2 (receptor interacting protein kinase 2) is recruited to NOD2 and becomes activated (via a tyrosine and serine phosphorylation and protein-dependent poly-ubiquitination) [26,27]. Active RIPK2 activates TAK1 (tank binding kinase 1) and subsequently IKK (IκB kinases) followed by the movement of the NF-κB dimer (p50 and p65) to the nucleus to turn on gene transcription [28,29,30,31,32].

Several lines of evidence indicate the important role of the NOD2/RIPK2 pathway in the pathogenesis of allergic airway inflammation [33], multiple sclerosis [34], granulomatous inflammatory disease [35], pancreatitis [36], psoriasis [37], sarcoidosis, and inflammatory bowel disease [38]. Thus, RIPK2 can play a role during inflammation injury in a number of disease settings, including cancer. In this study, we have explored the importance of RIPK2 in a systematic way in the origin and pathogenesis of IBC.

## 2. Results

### 2.1. IBC Cell Lines Exhibit High NF-κB Activity

**Figure 1:**
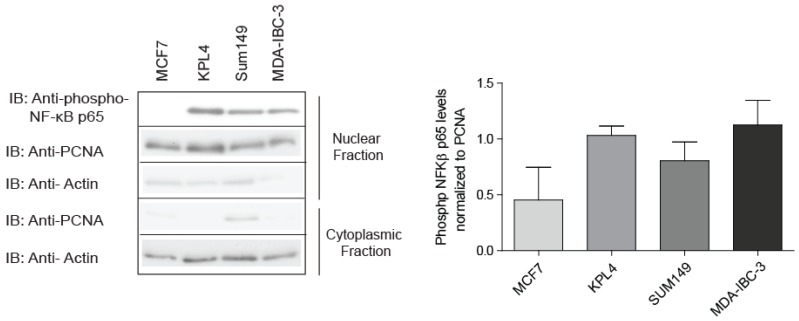
NF-κB (nuclear factor kappaB) activity in inflammatory breast cancer (IBC) cell lines. Equal concentrations of total protein from nuclear or cytoplasmic extracts were loaded into a gel and immunoblotted (IB) with the phospho-NF-κB p65 antibody that recognizes the p65 subunit phosphorylated at S536. PCNA (Proliferating cell nuclear antigen) expression is used as a control for nuclear fraction, and Actin is used as a control for cytoplasmic fraction. Signal was developed using enhanced chemiluminescence (ECL). Data shown are representative of the results of at least three independent experiments.

The involvement of NF-κB in IBC pathogenesis is more substantial than initially thought as studies have suggested that more than 50% of NF-κB associated genes are significantly upregulated in IBC in comparison to non-IBC patients [22]. In addition, NF-κB family members such as transcription factor Rel B and NFκB1 are elevated to a greater extent in IBC tumor tissue when compared to non-IBD, implying that NF-κB is constitutively active in IBC [21]. To confirm NF-κB activation in IBC cell lines, nuclear extracts from SUM149 cells, the most common cell line used to study IBC [39], MDA-IBC-3 and KPL4 were immunoblotted with a phospho-NF-κB p65 antibody detecting the phosphorylated site at serine 536 (S536). This specific site is phosphorylated by IKK and involved in the ability of p65 to transactivate gene expression resulting in NF-κB activation [40]. Indeed, nuclear extracts from IBC cell lines show high activation of NF-κB compared to luminal breast cancer cell line (MCF7) (Figure 1). This result was further supported by cytokine profiling using multiplex arrays, where IBC cell lines secreted significantly higher levels of NF-κB-promoting cytokine including IL-1β, IL-12, and INF-γ when compared to non-IBC cell lines [41,42,43]. These inflammatory cytokines are known to be abundantly produced by IBC tumor cells [42]. Besides being characterized as NF-κB activating stimuli [44,45], IL-1β increase is known to play a key role in tumor growth, survival, invasion, and metastasis, while INF-γ is involved in the regulation of cancer stem cells (CSCs) [23]. In addition, high levels of IL-12 are found in the serum of breast cancer patients and this correlates with tumor progression [46].

### 2.2. Elevated RIPK2 Activity Level in IBC Cell Lines and Patient Tissues

**Figure 2:**
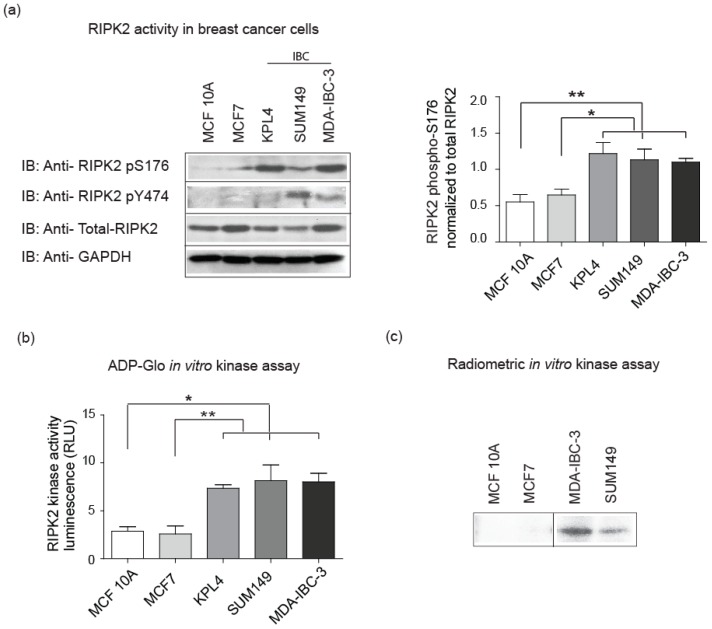
RIPK2 is hyperactive in IBC cell lines: (**a**) equal concentrations of samples were loaded and immunoblotted (IB) with an antibody to the active form of RIPK2 using a RIPK2 phospho (p) –Serine (S) 176, RIPK2 phospho (p) -tyrosine (Y) 474 or an antibody that recognizes total RIPK2. GAPDH (Glyceraldehyde 3-phosphate dehydrogenase) expression is used as a loading control for whole cell lysate. Signal was developed using enhanced chemiluminescence (ECL). * *p*-value for the difference between MCF 10A and KPL4, SUM149, and MDA-IBC-3 is 0.01, 0.02, and 0.004, respectively. ** *p*-value for the difference between MCF7 and KPL4, SUM149, and MDA-IBC-3 are 0.02, 0.03, and 0.001; (**b**) luminescent ADP-Glo in vitro RIPK2 kinase assay in breast cancer cell lines. RIPK2 was immunoprecipitated and kinase activity was then measured by quantifying luminescence (RLU) that correlates to the percentage of ADP produced during the enzymatic reaction as per manufacture instructions. * *p*-value for the difference between MCF 10A and KPL4, SUM149, and MDA-IBC-3 are 0.004, 0.09, and 0.04, respectively. ** *p*-value for the difference between MCF7 and KPL4, SUM149, and MDA-IBC-3 is 0.01, 0.03, and 0.02, respectively; and (**c**) radioactive in vitro kinase assay in different breast cancer cell lines (cut from the same gel). RIPK2 was immunoprecipitated and kinase activity was then determined using radioactively labeled ATP. The autophosphorylation site of Y474 of RIPK2 is visualized using standard autoradiography. All the data shown are representative of the results of two to four independent experiments. KPL4 revealed similar constitutively active level of RIPK2 [43].

It is currently unknown what are the molecular drivers of constitutively active NF-κB in IBC cells. RIPK2 is linked to NF-κB through upstream signaling receptors such as NOD2 or TNFα receptor [47,48]. RIPK2 expression is believed to be elevated in triple negative breast cancer (TNBC) and significantly correlated with worse progression in TNBC [47]. IBC cells exhibited high levels of active NF-κB when compared to non-IBC cells. We hypothesized that abnormalities in signaling proteins upstream from NF-κB, such as in RIPK2 may exist. Analysis of RIPK2 activation in IBC cell lines and patient tumor tissues revealed elevated kinase activity as determined by the surrogate detection tool, the use of a RIPK2 phospho antibody phosphorylated at Serine-176 (S176 indicative of active RIPK2) [27,49] and tyrosine-474 recognizing the autophosphorylation sites of RIPK2 (also indicative of active RIPK2) [50]. IBC cells lines show robust active RIPK2 when compared to non-IBC cell lines (Figure 2a). This activity was quantified using densitometry and all IBC cell lines revealed significantly increased level of RIPK2 activity when compared to MCF10A and MCF7 cell lines. Similar results were obtained using the RIPK2 ADP-Glo assay that measures the ADP formed from a kinase reaction and in a radiometric in vitro kinase assay that monitors the addition of ^32^P-γ-ATP to RIPK2 (Figure 2b,c).

**Figure 3:**
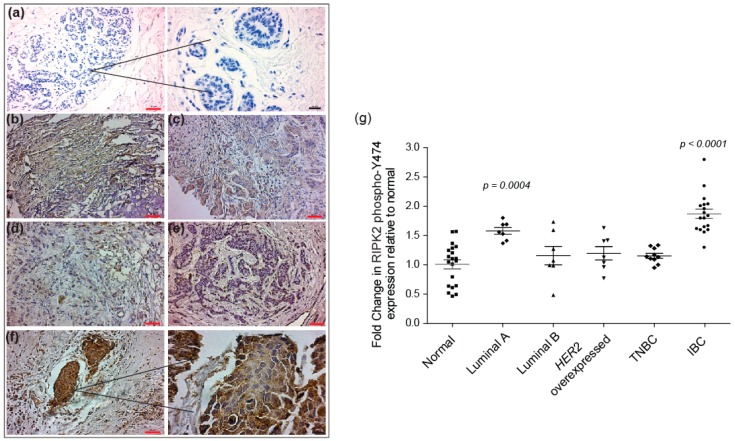
Immunohistochemical staining of normal non-neoplastic breast: (**a**) luminal A; (**b**) luminal B; (**c**) *HER2* overexpressed; (**d**) triple negative breast cancer (TNBC); (**e**) and IBC; (**f**) using RIPK2 phospho-Y474 antibody). Breast tissue was stained and visualized using horseradish peroxidase-conjugated secondary antibody and 3, 3′ diaminobenzidine (DAB; brown), red scale bar: 50 µm, black scale bar: 20 µm. DAB staining of luminal A (*n* = 7), luminal B (*n* = 8), *HER2* overexpressed (*n* = 7), TNBC (*n* = 10) and IBC (*n* = 18). Tissue was quantified using the ImageJ platform permitting integrated optical density assessment of regions of interested in each slide. ImageJ analyzed images were then normalized to normal breast tissue (*n* = 17) imaged in a similar manner; and (**g**) the plot represents the fold change in RIPK2 phospho-Y474 expression in tumor tissue relative to normal non-neoplastic breast tissues. *p*-value is calculated against normal breast tissue. All breast cancer tissues were isolated from patients after neoadjuvant chemotherapy treatment. A description of our patient population is documented in Table 1 (in the Materials and Methods Section 4.3).

In support of IBC cell line data, immunohistochemical (IHC) analysis using a RIPK2 phospho-Y474 antibody, IBC tissue revealed robust and diffuse positive cytoplasmic staining in invasive carcinoma versus non-neoplastic breast tissue (considered a normal control) (*p*-value *<* 0.0001) (Figure 3). Breast tissue of non-inflammatory breast cancer patients did not show a significant difference in RIPK2 activity compared to normal, Luminal B (*p*-value = 0.37), *HER2* overexpressed (*p*-value = 0.22), and TNBC (*p*-value = 0.22) except for Luminal A (*p*-value = 0.0004). However, this activity was still significantly less than IBC (*p*-value *<* 0.05), suggesting that the RIPK2 is highly activated in IBC tissue compared to non-IBC.

### 2.3. Neoadjuvant Chemotherapy Does Not Inhibit RIPK2 Activity

**Figure 4:**
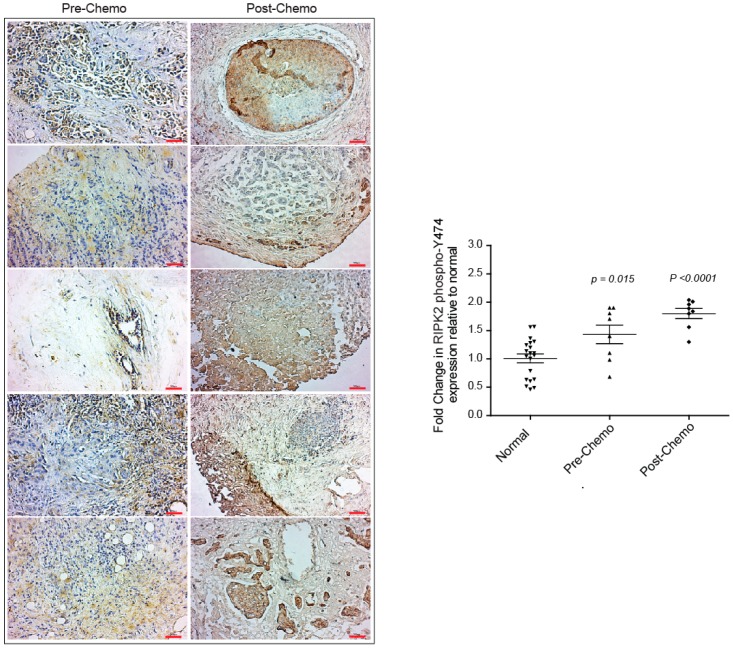
Immunohistochemical staining using the RIPK2 phospho-Y474 antibody in IBC breast tissue pre- and post-chemotherapy as indicated. Red scale bar: 50 µm. DAB staining was quantified using ImageJ software and normalized to normal non-neoplastic breast tissue. A total of eight IBC patient tissues pre- and post-chemotherapy were quantified. *p*-values represent the difference between normal and pre-chemo and post-chemo, respectively.

The evidence of active RIPK2 in IBC tumor tissues following neoadjuvant chemotherapy (such as treatment with three cycles of 5-fluorouracil, epirubicin, and cyclophosphamide [FEC] followed by three cycles of docetaxel) led us to question if RIPK2 was elevated in IBC patients at diagnosis and then was force-activated following chemotherapy. We were able to obtain eight tumor matched tissues of the same IBC patients at diagnosis and post-neoadjuvant chemotherapy and immmunohistochemically stained them with a RIPK2 phospho-Y474 antibody as a surrogate marker for active RIPK2. Surprisingly, RIPK2 activity was elevated in the pre-neoadjuvant chemotherapy tumor tissues when compared to normal (*p*-value = 0.006) and immunohistochemical staining was significantly elevated in matched post-chemotherapy tissue (*p*-value < 0.0001) (Figure 4). A significant difference of RIPK2 activity is still found between pre and post neoadjuvant chemotherapy (*p*-value < 0.05) (Figure 4). This result is not entirely surprising as increased inflammation following chemotherapy has often been reported [51]. Analysis of pre-chemotherapy tissue sections from IBC patients lead to two novel observations: (i) RIPK2 activity may be a contributory mediator to inflammation post-neoadjuvant chemotherapy and (ii) neoadjuvant chemotherapy may not necessarily lead to a decrease from the pre-chemotherapy levels of RIPK2 and thus may not be the ideal single mode of therapy to reduce inflammation and the proliferative effects of inflammation. Combination chemotherapy with a RIPK2 inhibitor, for example, might be a more targeted therapeutic approach for IBC patients.

### 2.4. RIPK2 Activity as an Independent Prognostic Marker

Since RIPK2 is highly activated in IBC, we wanted to assess its role as a prognostic marker. In breast cancer, The Tumor, node, metastasis (TNM) staging classification for breast cancer is a useful tool to evaluate prognosis of the disease [52]. According to the American Joint Committee on Cancer (AJCC)/International Union Against Cancer (UICC), TNM staging is based on the primary tumor size (T), the lymph nodes involvement (N) and the presence of distant metastases (M) [53]. Retrospective studies have indicated that TNM staging correlates with a patient’s survival rate in breast cancer [54,55].

**Figure 5:**
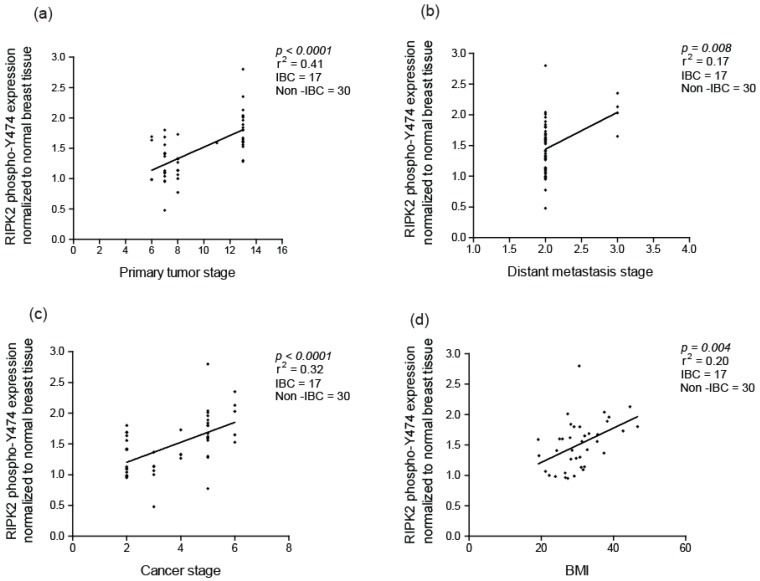
Correlation of active RIPK2 expression with: (**a**) primary tumor stage; (**b**) presence of distant metastasis stage; (**c**) cancer stage; and (**d**) body mass index (BMI) in breast cancer. Active RIPK2 denotes autophosphorylation at site Y474.

Using the Pearson correlation (r) coefficient, we found that RIPK2 activity correlated with primary tumor size stage (Slope 4.2, with a 95% CI of (2.6–5.8) and a *p*-value < 0.0001), the presence of distance metastasis (Slope 0.23, with a 95% CI of (0.06–0.39) and a *p*-value = 0.008) and cancer stage grouping (overall staging) (Slope 1.9, with a 95% CI of (1.06–2.7) and a *p*-value < 0.0001) (Figure 5a–c). However, RIPK2 activity was insignificantly associated with the number of lymph nodes involved (Slope 2.4, with a 95% CI of (−2.6–6.9) and a *p*-value = 1.1). These findings are supported by the association of NF-κB activity with cancer-specific survival (CSS) and tumor size in invasive ductal breast cancer [56]. In addition, RIPK2 expression is found to correlate to progression-free survival (PFS) in TNBC [47].

Furthermore, we also observed that RIPK2 activity strongly associates with a patient’s body mass index (BMI) (Slope is 7.2, with a 95% CI of (1.9–12.5) and a *p*-value = 0.003) (Figure 5d). Interestingly, BMI had an independent association with IBC in a study done at The University of Texas M.D. Anderson Cancer Center [57] and is considered one of the few risk factors identified in IBC. A plausible explanation for a role for BMI in activation of RIPK2 and inflammation could mainly be inflammation in adipose tissue promoting local and system inflammation in obese individuals [58,59]. The correlation of RIPK2 activity with advanced tumor size, metastasis status, cancer grouping stage and possibly BMI suggests that RIPK2 could be a marker for all breast cancer and specifically IBC progression.

## 3. Discussion

IBC etiology and pathogenesis is not fully understood. Tumor tissue of IBC patients is histopathologically similar to other subtypes of breast cancer, where it displays evidence of invasive carcinoma. However, its unique clinical manifestation makes “molecular inflammation” a potential hypothesis for its cause. Many studies have suggested that the activation of important inflammatory mediators such as NF-κB contributes to the aggressiveness and pathogenesis of IBC [19,20,21,22,23]. NF-κB is known to play a crucial role in tumor cell proliferation, differentiation, apoptosis, invasion, and metastasis [18,19,20,60]. RIPK2 is an upstream activator of NF-κB that is able to induce ubiquitination of NF-κB essential modulator. It plays an important function in recognizing intracellular bacterial infection through regulating and transducing NOD1 and NOD2 signaling [61].

Although RIPK2 has been extensively studied in other types of cancers especially colorectal cancer because of its link to NOD2 mutation, little is known about the NOD2-RIPK2 pathway in breast cancer.

**Figure 6:**
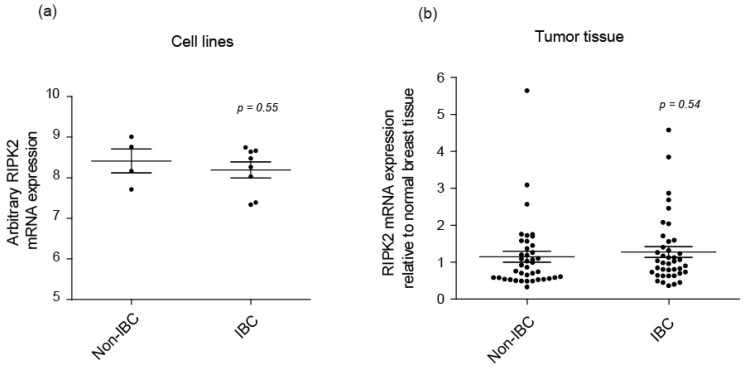
Total RIPK2 mRNA expression in Non-IBC and IBC: (**a**) cell lines (*n* = 12) GEO (Gene Expression Omnibus dataset) (GSE40464) [62] and (**b**) tumor tissue (*n* = 40) GEO dataset (GSE45584) [63] from public breast cancer expression array datasets. IBC cell lines include MDA-IBC-3, MDA-IBC-2, SUM149, and SUM190, non-IBC includes MDA-MB-231, MDA-MB-468. In (**b**) Non-IBC mainly refers to Luminal A, Luminal B, HER2 overexpressed, and TNBC.

Only one study has indicated that RIPK2 expression correlates with triple negative breast cancer [47]. Therefore, this is the first study to look at RIPK2 activity in IBC. In this study, we show that RIPK2 activity is increased in IBC cell lines and patient tissues by detecting the S176 and Y474 phosphorylation sites. This increase of activity was not relevant to the RIPK2 expression level in IBC as there was no significant difference in RIPK2 mRNA expression observed between IBC and non-IBC in cell lines (Figure 6a) and patient samples (Figure 6b) according to the GEO (Gene Expression Omnibus) datasets.

**Figure 7:**
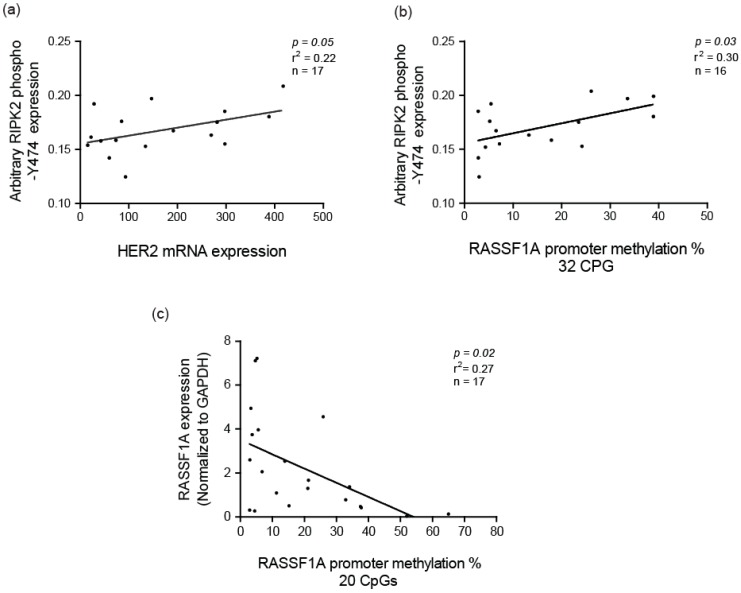
Correlation of active RIPK2 expression with HER2 mRNA expression: (**a**) and Ras association domain family protein 1A (*RASSF1A*) CpG methylation percentage; (**b**) correlation of *RASSF1A* mRNA expression and *RASSF1A* CpG methylation percentage; and (**c**) in IBC. CpG methylation analysis was carried out as described elsewhere [67] with focus on 32 CpG residues before the transcriptional start site (32 CpG *x*-axis label I (**b**) and CpG 13–32 (20 CpG) from the transcription start site.

Several studies have identified that NF-κB activation is correlated with HER2 status in breast cancer [56]. Hence, we decided to identify if HER2 mRNA expression correlated with RIPK2 activity in IBC patient samples. Interestingly, it did with parameters: (slope 0.24, with a 95% CI of (2.62×10^-6^–0.00015) and a *p* = 0.04) (Figure 7a), to suggest that HER2 might indirectly interact/influence RIPK2 activity in IBC. In fact, quantitative mass spectrometry-based proteomic and phosphoproteomic analyses of 105 breast cancer data have reported that RIPK2 has a similar gene amplification pattern to HER2 and that HER2 amplification showed an increase level of phosphoproteins [64]. Our result might be explained by the presence of an Erbb2 interacting protein (ERBB2IP) also known as Erbin. Erbin was found to be downregulated in Her2-overexpressing breast cancer cells [65], can form a complex with NOD2 (the obligate receptor for RIPK2) and work as a negative regulator of its activity [66]. It is plausible that the positive correlation between active RIPK2 and HER2 expression may be due to the Erbin downregulation in IBC and release of inhibition of NOD2/RIPK2.

Another explanation for increased activity of RIPK2 in IBC is the link to the tumor suppressor protein, Ras association domain family protein 1A (RASSF1A). Our research group has demonstrated that RASSF1A can restrict NOD2-RIPK2 association in intestinal cells to subsequently interfere with the ability of RIPK2 to drive NF-κB-directed molecular inflammation [43]. Epigenetic analysis via pyromark sequencing of 32 CpGs of the *RASSF1A* promoter region revealed that IBC patients have a higher percentage of CpG methylation in comparison to breast reduction surgery (BRS) patients (normal control) [67]. Similar to most solid cancers, high methylation of *RASSF1A* in IBC patients correlates with loss of expression [67]. We can observe a positive correlation between RIPK2 activity and methylation status of *RASSF1A* in IBC tumor samples (Figure 7b). Furthermore, the methylation status of RASSF1A negatively correlated with *RASSF1A* mRNA expression (Figure 7c) to suggest expression loss with increased levels of CpG methylation. Therefore, loss of *RASSF1A* in IBC may be an interesting marker for increased inflammation via hyperactive RIPK2 and increased growth (via reduced tumor suppression due to increased methylation of RASSF1A) in IBC.

RIPK2 is activated through the microbial recognition receptor NOD2 [26,27]. Therefore, the increase of RIPK2 activity in IBC can be expected since some studies have suggested IBC association with infectious agents. A study done at Ain Shams University in Egypt has indicated that a significant number of human cytomegalovirus (HCMV) DNA was detected in tumor tissue of IBC patients [41]. Another study identified human mammary tumor virus (HMTV) in more than 70% of Tunisian IBC patients and only 40% in non-IBC [68]. In addition, certain species of skin and intestinal bacteria have been isolated from mother’s milk during late pregnancy and lactation [69]. Though the source of this bacteria and mode of transition is still under study, this in line with the finding that breastfeeding increases the risk of IBC [70]. Altogether, this supports our finding of elevated RIPK2 activity in IBC tissue.

**Figure 8:**
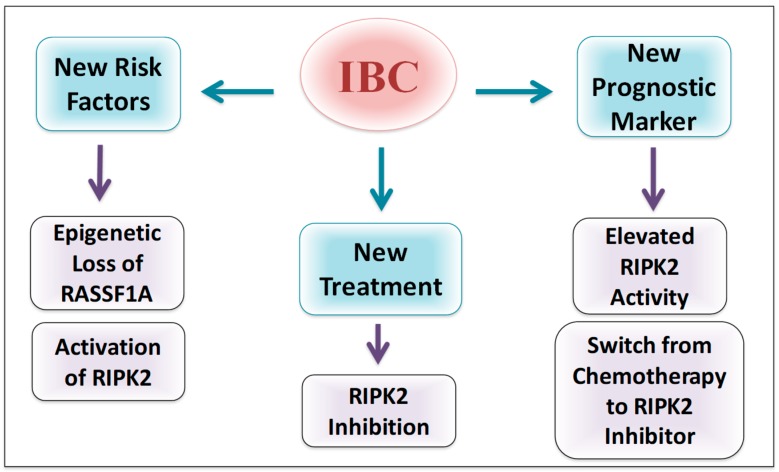
Potential importance of RIPK2 in IBC. IBC cell line and patient samples reveal elevated levels of active RIPK2 that may be linked to epigenetic silencing of RASSF1A. Loss of RASSF1A, therefore, could be a new risk factor in IBC while active RIPK2 may have a role in regulating cellular response to chemotherapy. We speculate that RIPK2 inhibitors may be emerging therapeutic options for IBC.

It is clear from our results that RIPK2 activity is constitutively activated in IBC with increased activity following chemotherapy treatment. The results also show a significant increase between pre-chemo and post-chemo tissue. However, more samples are needed in order to increase our sample size of pre/post chemotherapy samples. Chemotherapy-induced inflammation is common in cancer therapy and can lead to tumor-acquired resistance resulting in treatment failure and metastasis [45]. 5-fluorouracil, one of the chemotherapies used to treat a range of cancers including breast cancer, are now linked to NF-κB activation, pro-inflammatory cytokines increase and subsequently metastasis promotion [45]. The increase of NF-κB activity is associated with breast cancer chemoresistance [23,46,47,48]. Treatment resistance is one of the hallmarks of IBC resulting in the high rate of cancer recurrence [71,72,73]. Interestingly, the use of RNA interference (RNAi) showed that RIPK2 knockdown could significantly alter cancer cells chemosensitivity [49]. Together, pieces of evidence suggest the important role of RIPK2 in IBC chemoresistance and the potential success of RIPK2 inhibition in decreasing IBC recurrence (Figure 8).

The association we found between RIPK2 activity and advanced tumor size, metastasis status, cancer stage and BMI suggest that RIPK2 could be a prognostic marker for breast cancer and IBC progression (Figure 8). It is unclear for us why RIPK2 activity did not correlate with lymph node status but many studies have pointed out that there is no association between lymph node status and metastatic breast cancer such as in TNBC [74]. However, our model would suggest that epigenetic loss of RASSF1A is important to follow, especially after neoadjuvant therapy and may be utilized to predict early activation of RIPK2. Furthermore, since RIPK2 is a robust driver of inflammation in IBC tissues, it may be a good biomarker to monitor the effectiveness of neoadjuvant chemotherapy and be an interesting additional therapeutic option in HER2 negative breast cancer and as another co-therapy for patients that display HER2 overexpression.

## 4. Materials and Methods

### 4.1. Immunoblotting and Antibodies

Total cell lysates were performed by lysing cells in a 10× RIPA buffer containing 20 mM Tris-HCl (pH 7.5) 150 mM NaCl, 1 mM Na_2_ EDTA 1 mM EGTA 1% NP-40 1% sodium deoxycholate 2.5 mM sodium pyrophosphate 1 mM β-glycerophosphate 1 mM Na_3_ VO_4_ 1 µg/mL leupeptin and protease inhibitor mixture. Protein concentrations were then measured using Bio-rad protein assay protocol. Lysates are resolved on a 7.5% polyacrylamide gel and transferred to a polyvinylidene fluoride membrane. The following antibodies are used: from Santa Cruz (Dallas, TX, USA): RICK (H-300); RICK (A-10); PCNA Antibody (PC10); Actin (AC-15); ERK 1 Antibody (K-23); and ERK 2 Antibody (C-14). From cell signaling technology (Danvers, MA, USA): phospho-RIP2 (Ser176) (E1I9J) #14397; NF-κB p65 (D14E12) #8242; Phospho-NF-κB p65 (Ser536) (93H1) #4887. RIPK2 Phospho-Y474 polyclonal antibody was obtained from a MediMab production order from the Baksh lab (University of Alberta, Edmonton, AB, Canada). For immunoblots, densitometry was performed using the ImageJ software (National Institutes of Health, Bethesda, MD, USA) of the scanned image using region of interest analysis. All results were normalized to normal breast tissue.

### 4.2. Breast Cancer Samples

Normal non-neoplastic breast tissues from breast reduction surgery and breast cancer tissues were obtained from the Alberta Cancer Research BioBank (Edmonton, AB, Canada) with clinical data (formally the Canadian Breast Cancer Foundation tumor bank). All tissues were snap frozen within 30 min to preserve all the proteins. For most samples, tumor tissue was obtained, paraffin embedded and unstained slides generated for immunohistochemical analysis. Tissue samples are post-treatment unless specified. Patients are diagnosed and classified using TNM classification of the American Joint Committee on Cancer Normal non-neoplastic breast tissue from patients with and without breast carcinoma is provided by Cooperative Human Tissue Network (www.chtn.org). Pathological confirmation of breast cancer subtypes was carried out by Dr. Mehdi Agoumi and was based on morphology, overall histological grade (Notthingham Scoring system biomarkers expression (ER, PR, HER2 and KI-67) and clinical data as: Normal (Benign breast tissue with normal TDLU (Terminal duct lobular unit); Luminal A, Luminal B, Her2 overexpression, TNBC and IBC. Table 1 summarizes the characteristics of the breast cancer patients used in this study.

### 4.3. Cell Lines

The human IBC cell lines SUM149 were kindly provided by Dr. Lynne Postovit (University of Alberta) and were grown in Ham’s F12 medium supplemented with 5% FBS, 5 μg/mL insulin, and 1 μg/mL hydrocortisone. The human IBC cell lines KPL4 were kindly provided by Dr. Naoto T. Ueno (University of Texas MD Anderson Cancer Center, Houston, TX, USA) and were grown in Dulbecco’s modified Eagle’s medium/F12 medium supplemented with 10% FBS. The human IBC cell line MDA-IBC3 was kindly provided by Dr. Wendy Woodward’s laboratory (University of Texas MD Anderson Cancer Center) and were grown in Ham’s F12 medium supplemented with 10% FBS, 5 μg/mL insulin, and 1 μg/mL hydrocortisone. Non-IBC breast cancer cell lines MCF 10A, MCF7, MDA-MB-231 and BT549 were kindly provided by Dr. Mary Hitt (University of Alberta).

### 4.4. RIPK2 In Vitro Kinase Assay

Cells were lysed in 1× RIPK2 lysis buffer (50 mM Tris, pH 7.5, 10 mM MgCl_2_, 1% Triton X-100, 1 mM DTT, 1 mM EDTA, 1 mM EGTA, with freshly added 1 mM β-glycerophosphate and protease inhibitors (PMSF and aprotinin) and immunoprecipitated overnight using 1.5 mg rabbit anti-RIPK2 antibody. The next day, protein G-sepharose was used to immunoprecipitate the RIPK2 protein complex IP for 1.5 h. Samples were then washed once in 1× kinase wash buffer (50 mM Tris, pH 7.5, 150 mM NaCl, 1% Triton X-100, 1 mM EDTA) followed by 2 washes with 1× Kinase buffer (30 mM Hepes, pH 7.5, 10 mM MgCl_2_, 2 mM MnCl_2_). After last wash, 20 mL water was added to beads, followed by 1× Kinase buffer and 32P-γ-ATP and kinase reaction was allowed to proceed for 45–60 min at 30 °C. Protein loading dye was to the mix and samples were boiled and separated on an SDS-PAGE gel. The gel was dried and then exposed to X-ray film to capture the autophosphorylation of RIPK2.

### 4.5. RIPK2 ADP-Glo Kinase Assay (Promega)

Cells were lysed in 1× passive lysis buffer (Promega, Madison, WI, USA) and immunoprecipitated overnight using 1.5 mg rabbit anti-RIPK2 antibody. The next day, protein G-sepharose was used to immunoprecipitate the RIPK2 protein complex IP for 1.5 h. Samples were then washed once with 1× PBS followed with 2 washes with 5× kinase reaction buffer (40 mM Tris (pH 7.5), 20 mM MgCl_2_, and 0.1 mg/mL BSA). 10 μL of 1× kinase reaction buffer was then added and the mixture was incubated for 45–60 min at room temperature. Followed by the addition of 10 μL of ADP-Glo™ Reagent to terminates the kinase reaction and depletes any remaining ATP (40-min incubation time). ADP is then converted to ATP using 20 μL kinase detection reagent, which will generate light from the newly synthesized ATP using a luciferase/luciferin reaction (incubation is 60 min). The light generated is measured using ELISA (enzyme-linked immunosorbent assay) plate reader. The reading is proportional to the ADP present in the sample and the kinase activity.

### 4.6. Immunohistochemical and Immunoblot Staining and Evaluation

IHC staining was performed as described previously [66]. All IHC results were evaluated using modified ImageJ software platform using a script written by Dr. Gilbert Bigras in collaboration with Dr. Shairaz Baksh permitting integrated optical density assessment of regions of interest on each slide. The script, using color deconvolution, separates out the DAB “brown” stained and hemotoxyin “blue” stained areas to quantify the DAB antigen stained areas. For ImageJ quantitation, the region of interest selected was from the entire image which, in most cases, was the tumor and environment around the tumor.

### 4.7. Nuclear and Cytoplasmic Extracts

Nuclear and cytoplasmic fractions were prepared using the NE-PER Nuclear Cytoplasmic Extraction Reagent kit from ThermoFisher scientific (Waltham, MA, USA) according to the manufacturer’s instruction.

### 4.8. Statistical Analysis

Stata/ICI 13.1 [75] was used to perform all the statistical analysis and a *p* < 0.05 was considered for statistical significance. All *p*-values were calculated using a two-tailed *t*-test and experiments were carried out at least 2–4 times. Error bars in all graphs represent the standard error.

## 5. Conclusions

This is the first study that addresses RIPK2 activity increase in IBC compared to other types of breast cancer. We believe that the increase of RIPK2 activity is causing NF-κB activation and subsequently contributing to IBC pathogenesis and aggressiveness. It is still unclear what causes RIPK2 hyperactivation but we speculate that specific proteins such RASSF1A, Erbin, or infectious agents might also play a part in this activation. Our results lead us to suggest the possible use of RIPK2 as a prognostic tool for IBC progression and chemotherapy response. Lastly, we hypothesize that RIPK2 might be an effective target for treating IBC.

## Figures and Tables

**Table 1:** Characteristics of the breast cancer patients in this study.

Variable	*n* (%)	Variable	*n* (%)
Age		TNBC	
≤45	11 (22)	Yes	10 (20)
>45	39 (78)	No	40 (80)
Grade		Tumor Size	
I	11 (22)	≤3 cm	29 (58)
III	35 (70)	>3 cm	13 (26)
Unknown	4 (8)	Unknown	8 (16)
ER		PR	
Positive	20 (40)	Positive	28 (56)
Negative	27 (54)	Negative	19 (38)
Unknown	3 (6)	Unknown	2 (4)
TNM		HER2	
I	17 (34)	Positive	30 (60)
II	13 (26)	Negative	18 (36)
III	16 (32)	Unknown	2 (4)
IV	4 (8)		
