# Erratum: Zare, A. et al. RIPK2: New Elements in Modulating Inflammatory Breast Cancer Pathogenesis. *Cancers*, 2018, 10, 184

**DOI:** 10.3390/cancers10110425

**Published:** 2018-11-07

**Authors:** Alaa Zare, Alexandra Petrova, Mehdi Agoumi, Heather Armstrong, Gilbert Bigras, Katia Tonkin, Eytan Wine, Shairaz Baksh

**Affiliations:** 1 Department of Pediatrics, Faculty of Medicine and Dentistry, University of Alberta, 113 Street 87 Avenue, Edmonton, AB T6G 2E1, Canada; zare.alaa@gmail.com (A.Z.); apetrova@ualberta.ca (A.P.); harmstro@ualberta.ca (H.A.); wine@ualberta.ca (E.W.); 2 Anatomic Pathologist at DynalifeDx, Diagnostic Laboratory Services, Department of Laboratory Medicine and Pathology, University of Alberta, 113 Street 87 Avenue, Edmonton, AB T6G 2R3, Canada; mehdi.agoumi@fraserhealth.ca; 3 Cross Cancer Institute, Department of Laboratory Medicine and Pathology, University of Alberta, 11560 University Ave, Edmonton, AB T6G 1Z2, Canada; gilbert.bigras@albertahealthservices.ca; 4 Division of Medical Oncology, Department of Oncology, Faculty of Medicine and Dentistry, University of Alberta, Edmonton, AB T6G 2R7, Canada; katiaton@yahoo.com; 5 Division of Experimental Oncology, Department of Oncology, Faculty of Medicine and Dentistry, University of Alberta, 113 Street 87 Avenue, Edmonton, AB T6G 2E1, Canada; 6 Department of Biochemistry, Faculty of Medicine and Dentistry, University of Alberta, 113 Street 87 Avenue, Edmonton, AB T6G 2E1, Canada; 7 Cancer Research Institute of Northern Alberta, University of Alberta, Edmonton, AB T6G 2R7, Canada; 8 Women and Children’s Health Research Institute, Edmonton Clinic Health Academy (ECHA), University of Alberta, 4-081 11405 87 Avenue NW Edmonton, AB T6G 1C9, Canada

The authors wish to make the following correction to their paper [1]. The name of the fourth author was misspelt. The correct name should be “Heather Armstrong”. The email address of the third author was invalid. The correct email address should be “mehdi.agoumi@fraserhealth.ca”. The departmental affiliation for Alexandra Petrova is incorrect (Department of Biochemistry, Faculty of Medicine and Dentistry, University of Alberta, 113 Street 87 Avenue, Edmonton, AB T6G 2E1, Canada) and should be changed to “Department of Pediatrics, Faculty of Medicine and Dentistry, University of Alberta, 113 Street 87 Avenue, Edmonton, AB T6G 2E1, Canada”. The affiliation order was rearranged accordingly. The authors would like to apologize for any inconvenience caused. The change does not affect the scientific results. The manuscript will be updated and the original will remain online on the article webpage.
